# The Effectiveness of Thermal Stimulation Plus Conventional Therapy for Functional Recovery After Stroke: A Systematic Review and Meta-Analysis

**DOI:** 10.3390/jcm13226937

**Published:** 2024-11-18

**Authors:** Daniela Celi-Lalama, Aida Soria-Vizcaino, Lucía Fernanda Flores-Santy, Felipe Araya-Quintanilla, Wilmer Danilo Esparza, Iván Cuyul-Vásquez, Héctor Gutiérrez-Espinoza

**Affiliations:** 1 School of Physiotherapy, Faculty of Medical, Health and Life Sciences, International University of Ecuador (UIDE), Quito 170411, Ecuador; dacelila@uide.edu.ec; 2 Universidad Camilo José Cela, Villafranca del Castillo, 28692 Madrid, Spain; 3 Centro Neurohabilitar, Quito 170521, Ecuador; lorenasovi@hotmail.com; 4 Carrera de Fisioterapia, Pontificia Universidad Católica del Ecuador, Quito 170525, Ecuador; lfflores@puce.edu.ec; 5 Escuela de Kinesiología, Facultad de Odontología y Ciencias de la Rehabilitación, Universidad San Sebastián, Santiago 7510157, Chile; felipe.arayaq@uss.cl; 6 Facultad de Ciencias de la Salud y Bienestar Humano, Carrera de Medicina, Universidad Tecnológica Indoamérica, Ambato 180202, Ecuador; wilmeresparza@uti.edu.ec; 7 Departamento de Procesos Terapéuticos, Facultad de Ciencias de la Salud, Universidad Católica de Temuco, Temuco 4810399, Chile; ivancuyul@gmail.com; 8 Faculty of Education, Universidad Autónoma de Chile, Santiago 7500912, Chile

**Keywords:** stroke, thermal stimulation, rehabilitation, functional recovery, meta-analysis

## Abstract

**Background:** Motor impairments limit the functional abilities of patients after stroke; it is important to identify low-cost rehabilitation avenues. The aim of this study is to determine the effectiveness of thermal stimulation in addition to conventional therapy for functional recovery in post-stroke patients. **Methods:** An electronic search was performed in the MEDLINE, Scopus, Web of Science, EMBASE, CINAHL, SPORTDiscus, Epistemonikos, LILACS, and PEDro databases. The eligibility criterion was randomized clinical trials that analyzed the clinical effects of thermal stimulation plus conventional therapy. Two authors independently performed the search, study selection, data extraction, and risk of bias assessment. **Results:** Eight studies met the eligibility criteria, and six studies were included in the quantitative synthesis. For thermal stimulation plus conventional therapy versus conventional therapy alone, the mean difference (MD) for function was 6.92 points (95% CI = 4.36–9.48; *p* < 0.01), for motor function was 6.31 points (95% CI = 5.18–7.44; *p* < 0.01), for balance was 4.41 points (95% CI = −2.59–11.4; *p* = 0.22), and for walking was 1.01 points (95% CI = 0.33–1.69; *p* < 0.01). For noxious thermal stimulation versus innocuous thermal stimulation, the MD for activities of daily living was 1.19 points (95% CI = −0.46–2.84; *p* = 0.16). **Conclusions:** In the short term, adding thermal stimulation to conventional therapy showed statistically significant differences in functional recovery in post-stroke patients. The quality of evidence was high to very low according to GRADE rating. The studies included varied in the frequency and dosage of thermal stimulation, which may affect the consistency and generalizability of the results. A larger quantity and a better quality of clinical studies are needed to confirm our findings. PROSPERO registration: CRD42023423207.

## 1. Introduction

Stroke is the most common cause of disability in Western countries [1]. It is a disease that is considered to be a medical emergency with a high mortality rate [2]. A stroke can be classified as hemorrhagic, where there is a rupture of a blood vessel, or ischemic, where there is blood loss in a specific area. A hemorrhagic stroke is typically caused by arterial hypertension and is associated with high mortality rates [3]. In 2019, the incidence of this type of stroke increased by 70% and the prevalence by 85%, contributing to stroke being the second leading cause of death worldwide; these data are associated with the significant growth of the risk factors of high body mass index, ambient particulate matter pollution, and World Bank low-income group [4].

Typically, motor impairments limit the functional abilities of 80% of post-stroke patients [5]. Several studies indicate that hemiparesis is the most prevalent sequela after stroke in addition to limb spasticity, leading to neuromuscular control impairments, sensory alterations, cognitive deficits, and diminished motor function [1,6,7,8]. Furthermore, patients with upper-limb dysfunctions face significant challenges in performing daily living activities, such as dressing, eating, and selfcare [1,6,7,8]. This decrease in functional independence has a significant impact on the quality of life [9,10]. Consequently, upper-limb motor impairment has a longer recovery time than lower limb due to limitations in manual coordination and simultaneous motor tasks [11].

Generally, the treatment of stroke is multidisciplinary, where a physician uses common strategies to modulate spasticity with the application of botulinum toxin type A and intrathecal baclofen device implants, while rehabilitation after stroke focuses on the recovery of functional motor skills and sensory and cognitive alterations [10,12]. Physical rehabilitation and neurorehabilitation encompass therapeutic intervention that, historically, were predominantly based on empirical models lacking scientific evidence and offering limited variability in treatment approaches. However, these interventions are now widely implemented in clinical practice as a conventional therapy in post-stroke patients [10,13]. Several systematic reviews with or without meta-analysis have analyzed the clinical effects of neurorehabilitation interventions such as the neurodevelopment treatment, Bobath [10,14], therapeutic and proprioceptive exercises with proprioceptive neuromuscular facilitation [15], and motor learning [8,10].

Currently, there is significant variability in therapeutic intervention for conventional post-stroke conventional therapy, with many approaches aimed at enhancing neuroplasticity to improve functional outcomes [10]. Examples include immersive virtual reality [6], mobile applications [7], robotic rehabilitation [1,16], non-invasive brain stimulation [17], exercises for walking and activities of daily living [18], and thermal stimulation [10].

Interestingly, thermal stimulation using alternative hot and cold stimulation also activates several brain areas that regulate motor learning and create motor memories, similarly to the brain’s response when aerobic exercise or physical activity is performed [10]. Thermal stimulation sends electrical signals to the lateral spinothalamic tract, which induces the excitation of the sensory and motor cortex, facilitating neuroplasticity [19,20,21]. In line with this, some randomized clinical trials have reported significant improvement in motor function, balance, gait, and spasticity in patients post-stroke [9,19,20]. The evidence shows that thermal stimulation produces immediate neurophysiological changes in the motor cortex of the injured cerebral hemisphere and a significant increase in the size of cortical mapping in post-stroke patients [10,21,22,23].

To our knowledge, only two systematic reviews without meta-analysis have analyzed the clinical effectiveness of thermal stimulation in patients after stroke [22,23]. Thus, there is a need for a current synthesis of the evidence, as well to quantitatively analyze and calculate pooled estimate data related to the clinical effects of thermal stimulation in these patients. Therefore, the aim of this systematic review and meta-analysis was to determine the effectiveness of thermal stimulation in addition to conventional therapy for functional recovery in patients post-stroke.

## 2. Methods

### 2.1. Protocol and Registration

This study was performed according to Preferred Reporting Items for Systematic review and Meta-Analyses (PRISMA) [24]. The study protocol has been registered in the PROSPERO database with the registration number CRD42023423207.

### 2.2. Electronic Search

The electronic databases MEDLINE (via PubMed), Scopus, Web of Science (WoS), EMBASE, the Cumulative Index to Nursing and Allied Health Literature (CINAHL complete), SPORTDiscus, Epistemonikos, Latin American and the Caribbean Literature in Health Sciences (LILACS), and Physiotherapy Evidence Database (PEDro) were systematically searched from inception until 31 August 2024. The structure of the strategy was based on the PICOT acronym (population, intervention, comparison, outcomes, and study type) and followed the Cochrane Collaboration recommendation for developing sensitive search strategies [25]. The search strategy was designed using the National Library of Medicine’s controlled vocabulary (MeSH) in combination with free terms. The following MeSH and free terms were included in the search strategy: “Stroke” [Mesh], “Cerebrovascular disorders” [Mesh], (“Hyperthermia, Induced” [Mesh], ICTUS, Post stroke, After Stroke, Hemiplegic, Hemiparetic, Thermal intervention, Thermal stimulation, Thermotherapy, Thermal therapy, Thermal tactile stimulation, Thermotherapy, Innocuous thermal stimulation, Thermal approach. No filters or restrictions were used for any of the databases. Finally, the database searches were complemented with manual searches in the references of the included articles. The search strategy for each database is available in the Supplementary Material (Supplementary Table S1).

### 2.3. Eligibility Criteria

Studies on the clinical effectiveness of thermal stimulation in addition to conventional therapy in post-stroke patients were considered eligible for inclusion if the following criteria were fulfilled: (1) Population: patients older than 18 years who were clinically diagnosed with a first-ever stroke (no limits have been set on the type [ischemic or hemorrhagic], location [anatomical] of the lesion, or any degree of impairment severity post stroke). (2) Type of intervention: patients treated with a thermal stimulation program in addition to conventional therapy (i.e., neurorehabilitation interventions, therapeutics exercises, or other physical agents). Thermal stimulation is the use of heat at 46 °C to 48 °C and cold at an average of 7 °C to 8 °C alternately with a time interval of 15 to 30 s for 3 to 5 times per week for 6 to 8 weeks. There are two types of thermal stimulation: noxious thermal stimulation involves higher temperatures (46–47 °C for heat, 7–8 °C for cold) while innocuous thermal stimulation uses milder temperatures (40–41 °C for heat, 20–21 °C for cold) [26]. (3) Type of comparison: patients treated with conventional therapy such as different types of exercises, physical agents (i.e., ultrasound, TENS, or hot packs), neurorehabilitation interventions (i.e., neuro-developmental treatment, proprioceptive neuromuscular facilitation, or sensory integration), or occupational therapy. (4) Types of outcomes: the primary outcome was functional recovery assessed with upper or lower limb function questionnaires, and the secondary outcomes were balance, muscle tone, muscle strength, and range of motion. (5) Types of studies: controlled clinical trials or randomized clinical trials without restrictions on language.

The exclusion criteria were as follows: (1) studies involving patients with other neurological diseases (i.e., dementia, Alzheimer’s disease, epilepsy, or Parkinson’s disease); (2) studies involving patients with unstable cardiovascular or orthopedic diseases before stroke; (3) studies involving patients with sensory impairment attributable to peripheral vascular disease or neuropathy; (4) studies involving patients with a speech disorder or global aphasia; or (5) studies involving patients with contraindications of cold or heat (i.e., acute inflammation, venous thrombosis, or Raynaud’s disease).

### 2.4. Study Selection

Two reviewers (HG-E and IC-V) independently evaluated titles and abstracts in a standardized blinded way. We obtained the full text for all references that either author considered relevant for our systematic review. Inconsistencies were resolved by a consensus discussion. Unresolved disagreements were resolved by a third reviewer (DC-L). Additionally, the agreement rate between the reviewers was calculated using kappa statistics.

### 2.5. Data Collection Process

Two reviewers (LS-V and WD-E) independently extracted data from each trial. The following data were extracted from the original article: (1) authors and year of publication, (2) country, (3) sample characteristics (sample size, age, distribution, and sex), (4) characteristics of the thermal stimulation group, (5) characteristics of the conventional therapy or comparison group, (6) the length of follow-up and main outcomes, and (7) the main results. Additionally, the researchers contacted the corresponding authors to request information about missing and/or not reported data of the studies included.

### 2.6. Risk of Bias in Individual Studies

The risk of bias of the included studies was independently evaluated by two reviewers (FA-Q and LF-S) using the Risk of Bias 2 (RoB 2) tool proposal by Cochrane Collaboration [27]. This tool assesses the risk of bias of clinical trials according to the six domains: bias arising from the randomization process, bias due to deviations from intended interventions, bias due to missing outcome data, bias in measurements of the outcome, bias in selection of the reported result, and overall bias. Each domain could be considered as “low”, “some concerns”, or “high” RoB.

### 2.7. Statistical Methods

For statistical analysis, the DerSimonian and Laird random effect or Mantel–Haenszel fixed effect methods were used to compute a pooled estimate of the mean difference (MD) or standard mean difference (SMD) and respective 95% confidence intervals (CIs) [25,28]. The heterogeneity of the results across studies was evaluated using the I^2^ statistic [29], which considers 0 to 40% as negligible, 30 to 60% as moderate, 50 to 90% as substantial, and 75 to 100% as considerable heterogeneity [25]. The meta-analysis was performed with RevMan 5.4 program.

### 2.8. Rating the Quality of Evidence

The synthesis and quality of the evidence for each outcome were assessed using the Grading of Recommendation, Assessment, Development, and Evaluation (GRADE) approach [30]. The quality of the evidence was classified into four categories: high, moderate, low, and very low [31]. We used the GRADE profiler (GRADEpro) to import the data from RevMan 5.4 to create a “summary of findings” table. To categorize the quality of evidence, we considered six topics: (1) study design; (2) risk of bias of included studies; (3) inconsistency; (4) indirect evidence; (5) imprecision; and (6) publication bias and confounding factors.

## 3. Results

### 3.1. Study Selection

**Figure 1:**
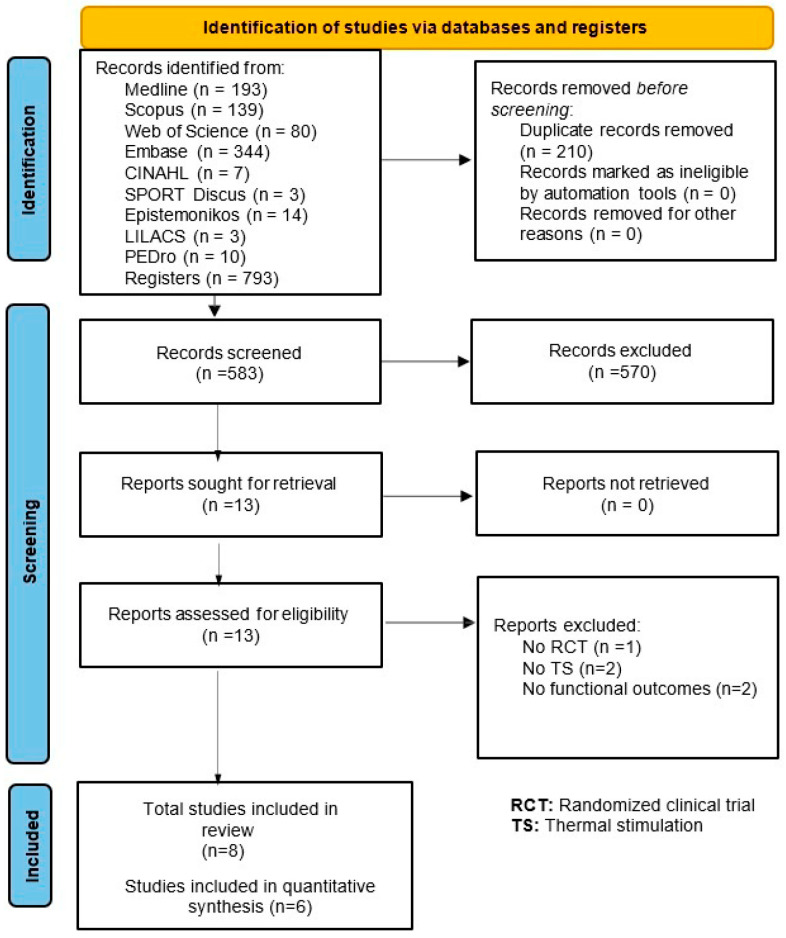
Flow chart diagram.

A total of 793 studies were found through the electronic searches (Figure 1). Eight studies met the eligibility criteria and were included in this systematic review [9,19,20,32,33,34,35,36]. No studies were found through the manual search. The kappa agreement rate between the reviewers was 0.92. In addition, excluded studies and the reasons for exclusion are available in the Supplementary Materials (Supplementary Table S2).

### 3.2. Study Characteristics

A summary of the included studies is presented in Table 1. The overall population included 301 patients (160 in the thermal stimulation plus conventional therapy group and 141 in the conventional therapy group). The mean age for the thermal stimulation plus conventional therapy group was 57.5 years, and it was 57.2 for the conventional therapy group. Additionally, the included studies were classified into the following comparisons: (1) thermal stimulation plus conventional therapy versus conventional therapy [9,19,20,32,33,36] and (2) noxious thermal stimulation plus conventional therapy versus innocuous thermal stimulation plus conventional therapy [34,35].

Regarding the treatment frequency and dose, four included studies conducted treatment five times per week for four to six weeks [19,33,35,36], and the other four studies conducted treatment three times per week for eight weeks [9,20,32,34]. Five studies applied two cycles of thermal stimulation that included 15 s of heat stimulation and 30 s of cold stimulation [19,20,32,34,35], with 15 to 30 s rest period between stimulation. Conversely, two studies applied three cycles of thermal stimulation that included 30 s of heat stimulation, 30–45 s of cold stimulation, and a 30 s rest period between stimulations [9,33]. The last study applied eight rounds of 30 s each one (hot and cold stimulation) without a rest between stimulations [36].

### 3.3. Risk of Bias Assessment in the Individual Studies

**Figure 2:**
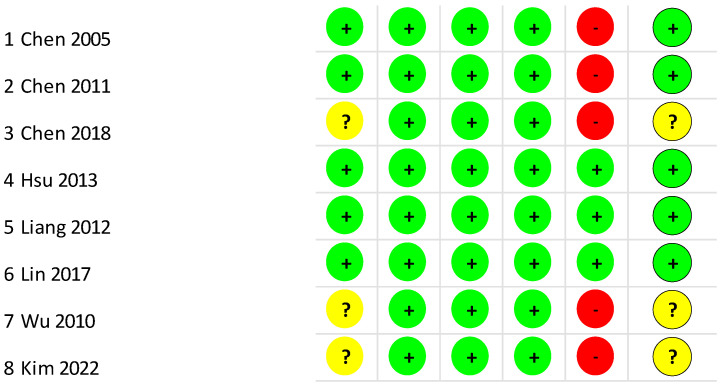
Risk of bias summary: review authors’ judgements about each risk of bias item for included study. Note: “low” (green ball or “+”) “unclear” (yellow ball or “?”) and “high” (red ball or “-“) risk of bias [9,19,20,32,33,34,35,36].

**Figure 3:**
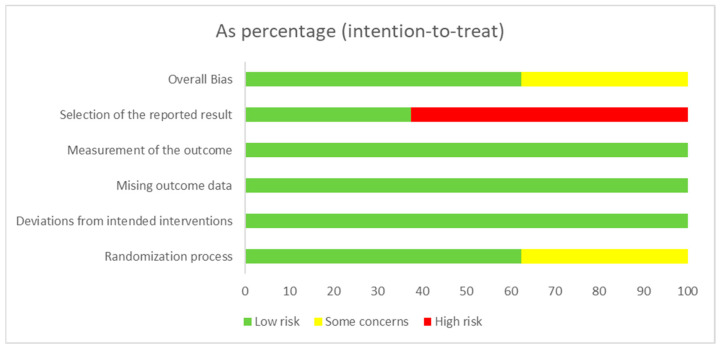
Risk of bias graph: review authors’ judgments about each risk of bias item presented as percentages across all included studies.

The RoB 2 assessment for all clinical studies is presented in Figure 2 and Figure 3. For the overall bias, 62.5% of the studies were scored as “low risk” of bias [9,19,33,34,35], and 37.5% were scored as “some concerns” [20,32,36]. For the randomization, 62.5% of the clinical studies were scored as “low risk” [9,19,33,34,35]. For the missing outcome data, 100% of the studies were scored as “low risk” [9,19,20,32,33,34,35,36]. For the measurement of the outcome, 100% of the studies were scored as “low risk” [9,19,20,32,33,34,35,36]. Finally, for the selection of the reported results, 62.5% of the studies were scored as “high risk” [9,19,20,32,36].

### 3.4. Publication Bias

Publication bias was evaluated by visual inspection of the funnel plots and the method proposed by Egger [37,38].

### 3.5. Synthesis of Results

#### 3.5.1. Thermal Stimulation Plus Conventional Therapy vs. Conventional Therapy

##### Lower Limb Function

**Figure 4:**
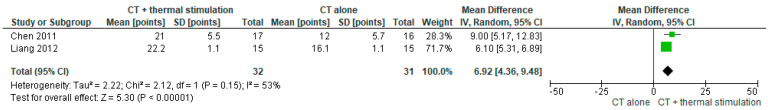
Forest plot of lower limb function at 6 weeks. CT: conventional therapy [9,33].

Two studies included data used to perform a meta-analysis of lower limb function at six weeks, measured with the Fulg Meyer assessment scale for lower extremity (FMA-LE) [9,33]. There was a statistically significant difference in the overall pooled data (MD = 6.92 points; 95% CI = 4.36–9.48; *p* < 0.01) in favor of the thermal stimulation plus conventional therapy group (Figure 4), with moderate heterogeneity (I^2^ = 53%, *p* = 0.15). There was a high quality of evidence according to the GRADE rating. These data show that adding thermal stimulation to conventional therapy improves lower limb function.

##### Motor Function

**Figure 5:**
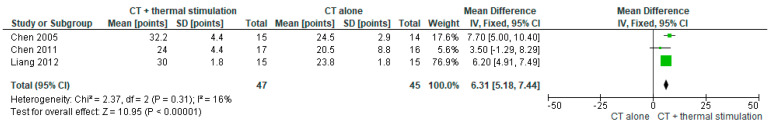
Forest plot of motor function at 6 weeks. CT: conventional therapy [9,19,33].

Three studies included data used to perform a meta-analysis of motor function at six weeks, measured with the Modified Motor Assessment Scale (MMAS) [9,19,33]. There was a statistically significant difference in the overall pooled data (MD = 6.31 points; 95% CI = 5.18–7.44; *p* < 0.01) in favor of the thermal stimulation plus conventional therapy group (Figure 5), with unimportant heterogeneity (I^2^ = 16%, *p* = 0.31). There was a high quality of evidence according to the GRADE rating. Alterations in motor function are related to the lack of independence that limits the quality of life after a stroke. These data show that adding thermal stimulation to conventional therapy improves motor function.

##### Balance

**Figure 6:**
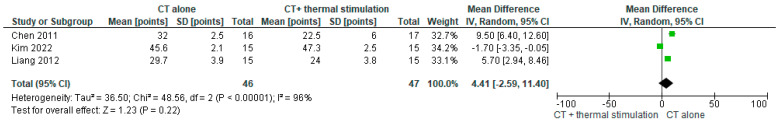
Forest plot of balance at 6 to 8 weeks. CT: conventional therapy [9,33,36].

Three studies included data used to perform a meta-analysis of balance at six to eight weeks, measured with the Berg Balance Scale (BBS) [9,33,36]. There was not a statistically significant difference in the overall pooled data (MD = 4.41 points; 95% CI = −2.59–11.4; *p* = 0.22) (Figure 6), with considerable heterogeneity (I^2^ = 96%, *p* < 0.01). There was a very low quality of evidence according to the GRADE rating. These data show that adding thermal stimulation to conventional therapy does not improve balance in these patients.

##### Walking

**Figure 7:**
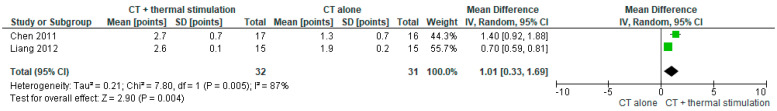
Forest plot of walking at 6 weeks. CT: conventional therapy [9,33].

Two studies included data used to perform a meta-analysis of walking at six weeks, measured with the Functional Ambulation Classification (FAC) [9,33]. There was a statistically significant difference in the overall pooled data (MD = 1.01 points; 95% CI = 0.33–1.69; *p* < 0.01) in favor of the thermal stimulation plus conventional therapy group (Figure 7), with moderate heterogeneity (I^2^ = 87%, *p* < 0.01). There was a low quality of evidence according to the GRADE rating. These data show that adding thermal stimulation to conventional therapy improves walking.

#### 3.5.2. Noxious Thermal Stimulation Plus Conventional Therapy vs. Innocuous Thermal Stimulation Plus Conventional Therapy

##### Activities of Daily Living

**Figure 8:**
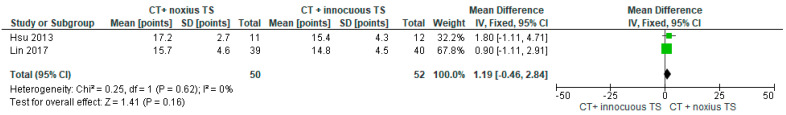
Forest plot for activities of daily living at 4 to 8 weeks. CT: Conventional therapy; TS: Thermal stimulation [34,35].

Two studies included data used to perform a meta-analysis for activities of daily living function at four to eight weeks, measured with the Barthel Index (BI) [34,35]. There was not a statistically significant difference in the overall pooled data (MD = 1.19 points; 95% CI = −0.46–2.84; *p* = 0.16) (Figure 8), with unimportant heterogeneity (I^2^ = 0%, *p* = 0.62). There was a high quality of evidence according to the GRADE rating. In the activities of daily living, there was no specific benefit to noxious or innocuous thermal stimulation.

The overall quality and summary of evidence with the GRADE approach are presented in Table 2.

#### 3.5.3. Publication Bias

The results of publications bias did not identify publication bias (Egger test; *p* = 0.532). See Supplementary Materials (Figure S1).

## 4. Discussion

This study aimed to determine the clinical effectiveness of thermal stimulation in addition to conventional therapy for functional recovery in patients after stroke. The main findings were that adding thermal stimulation to conventional therapy showed statistically significant short-term differences in lower limb function, motor function, and walking. Conversely, noxious thermal stimulation compared with innocuous thermal stimulation did not show statistically significant differences in the activities of daily living.

Most subjects that have suffered a stroke present deficits in motor function, which is why balance decreases and, with it, the ability to walk [39]. Regarding the upper limbs, the manipulation and grasping of objects is affected, since the movements are slow and less precise [40]. These clinical manifestations have a greater link to daily life activities, generating the highest disability in Western countries [1]. According to this, the application of thermal stimulation shows immediate neurophysiological changes in the motor cortex of the injured hemisphere in patients with stroke [35]. A significant increase in the size of the cortical mapping and the evoked potentials of some muscles relevant to the tasks motor execution could be the mechanism that explains the favorable results in walking, balance, spasticity, and functionality in these patients [20].

In line with our findings, two previous systematic reviews without meta-analysis have shown that thermal stimulation combined with conventional therapy facilitated motor recovery in acute and subacute stroke patients [22,23]. Thermal stimulation improved several aspects of the upper- and lower-limb function post-stroke when applied in the early stages of rehabilitation. Its clinical benefits have been observed short-term and were maintained at 3-month follow-ups, but disappeared at 6-month follow-ups [10,22]. The findings of our meta-analysis show that in the short-term, thermal stimulation may enhance the effects of conventional therapy and may be an effective therapeutic intervention when combined with conventional therapy in acute and subacute patients.

Regarding the addition of thermal stimulation to conventional therapy, this meta-analysis showed statistically significant benefits for functional recovery post-stroke. Several mechanisms of neuronal plasticity could be explained in our findings. Thermal stimulation enhances corticomotor excitability, and this intervention facilitates the sensory–motor interaction through the activation of skin thermoreceptors, which stimulate the spinothalamic pathway, improving cortical and ascending synapses [41]. Moreover, it stimulates brain activity in somato-sensory and motor areas at the cortical level, and this simultaneous activation of several brain areas could promote a restoration of brain functions and favor motor recovery [26]. Additionally, it can induce the release of neurotrophic and nerve growth factors involved in neuronal plasticity, neurogenesis, and memory and learning, similarly to physical activity [26,42].

Current evidence has proposed that thermal stimulation-induced cortical excitability functions differently depending on the noxious and innocuous thermal stimulus [43]. In this sense, innocuous stimulation stimulates thermal receptors, activating the primary and secondary somatosensory cortex and thalamus, while noxious stimulation stimulates nociceptors and effectively activates the lateral and medial pain systems involving the motor association areas, including the supplementary motor cortex and the anterior cingulate cortex [44,45]. The insula have a relevant brain-processing role in relation to noxious stimuli; the anterior insula is related to affective and cognitive components related to pain, and the posterior insular cortex to the discriminative sensory processing of thermal stimuli, thus relating with cortical brain activity and leading to an expansion of motor maps [21,46,47,48]. Despite this, our meta-analysis did not find significant differences in the activities of daily living evaluated through the BI. We believe that these results are due to activities of daily living not being directly related to motor patterns, but rather to levels of independence. Indeed, better neuromuscular control does was not associated with more independence, and the elements evaluated by the BI, such as the evaluation of bathing, grooming, eating, dressing, and transfers, do not reflect the motor function of post-stroke patients [49].

Interestingly, other factors that could affect our results were the variability in the treatment frequency and dosage of thermal stimulation. Four studies conducted treatment five times a week for a period of four to six weeks [19,33,35,36]. Four of these studies used innocuous thermal stimulation; therefore, it is assumed that innocuous stimulation is applied more frequently, but with a shorter treatment time period [9,19,33,36]. Three studies carried out the intervention three times a week for eight weeks, and all used noxious thermal stimulation [20,32,34]. In the literature, as a reference, it is established that noxious heat stimulation is on average 46–47 °C, with cold extremes of 7.8–3.3° C, and that innocuous heat stimulation is 40–41 °C, with cold extremes of 20–21 °C [21,48,50]; however, temperature measurement is a major limitation for this therapeutic intervention, since most studies used hot and cold compresses with the initial temperature intensity probably being lost in each cycle of application of thermal stimulation.

The clinical implications of this meta-analysis highlight the use of thermal stimulation in addition to conventional therapy, which provides significant short-term improvements in lower limb function, motor control, and walking ability in post-stroke patients. This suggests that clinicians should consider incorporating thermal stimulation early into the rehabilitation process to enhance motor recovery. Although thermal stimulation improves motor functions in the short term, it does not appear to significantly improve independence in daily living activities. This indicates that better motor control does not necessarily translate into greater independence in everyday tasks, underscoring the need for complementary therapies targeting functional independence. Regarding the differential effect of noxious or innocuous thermal stimuli, clinicians should choose between these depending on the desired therapeutic outcomes. While noxious stimulation engages motor association areas and pain systems, innocuous stimulation focuses on sensory processing. Finally, thermal stimulation is a low-cost, non-invasive therapy that can be easily incorporated into existing rehabilitation protocols across various settings, including home-based and outpatient care. Its ease of application and patient comfort make it a practical adjunct to conventional stroke rehabilitation [51,52].

To the best of our knowledge, this is the first meta-analysis to analyze the effects of thermal stimulation for functional recovery in post-stroke patients. Based on the PRISMA guidelines, the recommendations of the Cochrane Collaboration Handbook, the synthesis and quality of evidence assessed with GRADE, and the registration of the protocol in PROSPERO, this study used a transparent method for assessing and reporting the evidence.

### Limitations

The limitations of our study are as follows: (1) although we searched nine databases, we could have missed articles relevant to our search; (2) methodological limitations, such as the lack of an adequate sample size, unclear concealed allocation, and the lack of blinding of patients and assessors, could overestimate the effect size of the interventions studied; (3) the variability in the interventions, doses, and outcome measures in the included studies could be responsible for the high levels of heterogeneity reported; (4) due to the limited number of studies that met the eligibility criteria, the forest plots performed included few studies (two or three trials); (5) few studies evaluated the clinical effectiveness of thermal stimulation at medium- or long-term follow-up; and (6) in the planning stages, we intended to conduct subgroup analyses based on age, stroke stage (acute, subacute, or chronic), or affected upper or lower limb; however, this was not possible due to lack of data availability. Finally, our findings should be interpreted with caution in relation to the methodological limitations, few studies included in the quantitative synthesis, high heterogeneity of included studies, and limited strength of the available evidence.

## 5. Conclusions

In the short term, adding thermal stimulation to conventional rehabilitation showed statistically significant differences in functional recovery in post-stroke patients. The quality of evidence was high to very low according to GRADE rating. The studies included varied in the frequency and dosage of thermal stimulation, which may affect the consistency and generalizability of the results. This variability underscores the need for standardized treatment protocols to optimize outcomes and inform clinical guidelines. Despite this, we believe that physiotherapists should incorporate thermal stimulation for the clinical management of these patients; however, a larger quantity and a better quality of clinical studies are needed to confirm our findings.

## Figures and Tables

**Table 1:** Characteristics of the included studies.

Author	Country	Population		Intervention		Outcomes	Follow-Up	Results
		Sample Size (*n*)	Patients Mean (SD)	Intervention	Characteristics/Dose			
Chen et al., 2005 [19]	Taiwan	CG: 14 EG: 15	CG: 59.6 (12.0) EG: 58.5 (12.9) Patients with stroke <1 month	CG: Conventional therapy plus education. EG: Conventional therapy plus TS upper extremity.	CG: Conventional therapy (electromyographic biofeedback, active neuromuscular stimulation, acupuncture-like electrical stimulation and sensorimotor stimulation) for 6 weeks. EG: Conventional therapy plus TS intervention 30 sessions of 20–30 min duration 5 times weekly for 6 weeks. Each session of TS contained alternate cycles of 15 s of heating (48.8 ± 0.3 °C) and 30 s of cold (14.0 ± 0.2 °C) with ≥30 s pause.	-MMAS -Brunnstrom stage -Grasping -Wrist flexion -Wrist extension -Sensation	6 weeks	-MMAS **(*p* = 0.001)** -Brunnstrom stage **(*p* = 0.005)** -Grasping (*p* = 0.019) -Wrist flexion (*p* = 0.007) -Wrist extension **(*p* = 0.01)** -Sensation **(*p* = 0.02)**
Wu et al., 2010 [32]		CG: 11 EG: 12	CG: 54.3 (10.3) EG: 59.9 (11.4) Patients with stroke onset >3 months and <3 years.	CG: Conventional therapy plus TS on lower extremity. EG: Conventional therapy plus TS on the upper extremity.	CG: Conventional therapy (physiotherapist and occupational therapy) plus TS intervention 24 sessions with alternate cycles of heating (46–47 °C) and cold (7–8 °C) on lower limb for 3 times per week for eight weeks. EG: Conventional therapy plus TS intervention 24 sessions with alternate cycles of heating (46–47 °C) and cold (7–8 °C) on upper limb for 3 times per week for eight weeks.	-UE-STREAM -ARAT -LE-STREAM -BI -MAS	8 weeks/1 month	-UE-STREAM **(*p* = 0.002)** -ARAT **(*p* = 0.009)** -LE-STREAM (*p* = 0.16) -BI (*p* = 0.003) -MAS elbow **(*p* = 0.003)** -MAS wrist **(*p* = 0.01)**
Chen et al., 2011 [9]	Taiwan	CG: 16 EG: 17	CG: 62.3 (11.3) EG: 58 (11.5) Patients with stroke <1 month	CG: Conventional therapy plus education. EG: Conventional therapy plus TS.	CG: Conventional therapy (techniques of rehabilitation neurologic) 3 times per week. EG: Conventional therapy plus TS intervention 30 sessions with alternate cycles of 30 s of heating (46.5 °C) and 45 s of cold (15.5 °C) Eight repetitions of TS followed by 30 s of rest.	-FMA-LE -MRC-LE -MMAS -PASS -BBS -FAC -IW	6 weeks	-FMA-LE **(*p* = <0.001)** -MRC-LE **(*p* = <0.001)** -MMAS **(*p* = 0.010)** -PASS (*p* = 0.597) -BBS **(*p* = 0.007)** -FAC **(*p* = <0.001)** -IW (*p* = 0.057)
Liang et al., 2012 [33]	Taiwan	CG: 15 EG: 15	CG: 59.73 (11.6) EG: 56.1 (11.9) Patients with stroke <4 weeks	CG: Conventional therapy plus education. EG: Conventional therapy plus TS.	CG: Conventional therapy (Intensive exercise or cycling exercise, functional electrical stimulation, body weight support treadmill and robotic gait trainer) 3 times per 6 weeks, 18 sessions. EG: Conventional therapy plus TS intervention 30 sessions of 48 min. Alternate cycles of 30 s of heating (46.5 °C) and 45 s of cold (15.5 °C) 5 times a week for 6 weeks.	-FMA-LE -MRC-LE -FAC -BBS -MMAS -BI	6 weeks/3 months/6 months/1 year	At 3 months -FMA-LE 5.1 **(*p* = 0.000)** -MRC-LE 3.5 **(0.01)** -FAC 4.4 **(*p* = 0.005)** -BBS 3.0 **(0.038)** -MMAS 3.2 **(0.026)** -BI 3 **(0.031)** At 6 months -FMA-LE 4.4 **(*p* = 0.001)** -MRC-LE 2.5 (0.07) -FAC 3.0 (*p* = 0.058) -BBS 2.7 (0.054) -MMAS 2.3 (0.225) -BI 2.4 (0.089) At a 1 year -FMA-LE 3.5 **(*p* = 0.013)** -MRC-LE 1.8 (0.2) -FAC 1.6 (*p* = 0.013) -BBS 2.6 (0.132) -MMAS 1.1 (0.429) -BI 1.5 (0.148)
Hsu et al., 2013 [34]	Taiwan	CG: 17 EG: 17	CG: 52.6 (13.3) EG: 51.1 (14) Patients with stroke >3 month and <1 year	CG: Conventional therapy plus innocuous TS. EG: Conventional therapy plus noxious TS.	CG: Conventional therapy (physical and occupational therapy focused on functional task practice) plus innocuous TS intervention 24 sessions in lower limb with alternate cycles of heating (46–47 °C) and cold (23–24 °C) 3 times per week for 8 weeks. EG: Conventional therapy plus noxious TS intervention 24 sessions in lower limb with alternate cycles of heating (46–47 °C) and cold (2–3 °C) 3 times per week for 8 weeks.	-LE-STREAM -MOB-STREAM -FAC -BI -PASS -MAS	8 weeks/12 weeks	-LE-STREAM **(*p* = 0.028)** -MOB-STREAM **(*p* = 0.043)** -FAC (*p* = 0.073) -BI **(*p* = 0.013)** -PASS (*p* = 0.276) -MAS **(*p* = 0.034)**
Lin et al., 2017 [35]	Taiwan	CG: 40 EG: 39	CG: 61.8 (12.8) EG: 61.3 (12.0) Patients with stroke <1 month	CG: Conventional therapy plus innocuous TS. EG: Conventional therapy plus noxious TS.	CG: Conventional therapy plus innocuous TS intervention 20 to 24 sessions with alternate cycles of heating (40–41 °C) and cold (20–21 °C) for 30 min once per day. EG: Conventional therapy plus noxious TS intervention 20 to 24 sessions with alternate cycles of heating (46–47 °C) and cold (7–8 °C) for 30 min once per day.	-FMA-UE -ARAT -MI -BI -MAS	4 weeks/1 month/6 months	1 month -FMA-UE (*p* = 0.02) -ARAT (*p* = 0.002) -MI (*p* = 0.02) -BI (*p* = 0.01) -MAS (*p* = 0.02) 6 months -FMA-UE (*p* = 0.01) -ARAT **(*p* = <0.001)** -MI (*p* = 0.05) -BI (*p* = 0.02) -MAS (*p* = 0.02)
Chen et al., 2018 [20]	Taiwan	IG1: 13 IG2: 13 IG3: 17	IG1: 55.7 (14.0) IG2: 61.9 (11.0) IG3: 55.1 (16.7) Patients with stroke <6 months	IG1: NMES plus conventional therapy. IG2: TS plus conventional therapy. IG3: TS, NMES, plus conventional therapy.	IG1: NMES plus conventional therapy (stretching, endurance and strengthening exercises) 24 sessions. IG2: Conventional therapy (stretching, endurance and strengthening exercises) plus TS intervention 24 sessions with alternate cycles of heating (47 ± 1 °C) and cold (7 ± 1 °C). For 8 weeks, 3 times per week. IG3: Conventional therapy, NMES plus TS intervention 24 sessions with alternate cycles of heating (47 ± 1 °C) and cold (7 ± 1 °C). For 8 weeks, 3 times per week.	-FMA-UE -MI -MAS -BI	4 weeks	-FMA-UE (*p* = 0.49) -MI (*p* = 0.73) -MAS (*p* = 0.29) -BI (*p* = 0.71)
Kim et al., 2022 [36]	Korea	CG: 15 EG: 15	CG: 54.1 (9.3) EG: 53.2 (10.1)	CG: Conventional therapy. EG: Conventional therapy and TS lower extremity.	CG: Conventional therapy (proprioceptive neuromuscular facilitation, Bobath neurodevelopment therapy, balance training, gait training, muscle strengthening exercises), 40 sessions. EG: Conventional therapy plus TS intervention 40 sessions, with alternate cycles of heating (45–48 °C) and cold (11–15 °C) for 15 min. For 8 weeks, 5 times per week.	-TIS -COP -LOS -BBS -FRT -ASL -ASS -GC -Cadence −10 m W/T	8 weeks	-TIS **(*p* = 0.001)** -COP **(*p* = 0.001)** -LOS **(*p* = 0.001)** -BBS **(*p* = 0.000)** -FRT **(*p* = 0.005)** -ASL **(*p* = 0.001)** -ASS **(*p* = 0.004)** -GC **(*p* = 0.001)** -Cadence **(*p* = 0.044)** −10 m W/T **(*p* = 0.003)**

**SD:** standard deviation; **CG:** control group; **EG**: experimental group; **TS**: thermal stimulation; **MMAS:** Modified Motor Assessment Scale; **UE-STREAM**: up extremity subscale of stroke rehabilitation assessment of movement; **ARAT:** action research arm test; **LE-STREAM**: low extremity subscale of the stroke rehabilitation assessment of movement **BI:** Barthel index; **MAS:** modified Ashworth scale; **FMA-LE:** Fulg Meyer assessment—low extremity; **MRC-LE:** Medical research council scale—Low extremity; **PASS:** postural assessment scale for stroke patients; **BBS**: Berg balance score; **FAC:** Functional ambulatory category; **IW**: Independence walk; **MOB-STREAM**: mobility subscale of the stroke rehabilitation assessment of movement; **MI:** motricity index: **NMES:** neuromuscular electrical stimulation; **TIS:** Trunk impairment scale; **COP:** center of pressure; **LOS:** limit of stability; **FRT:** functional reach test; **ASL:** affected step length; **ASS:** affected single support; **GC:** gait cycle; **10 m WT:** 10 m walking test; **in bold**: statistically significant differences.

**Table 2:** Summary of findings (SoF) and quality of evidence (GRADE) for thermal stimulation plus conventional therapy versus conventional therapy alone.

Certainty Assessment							No. of Patients		Effect		Quality of Evidence (GRADE)	Importance
No. of Studies	Study Design	Risk of Bias	Inconsistency	Indirectness	Imprecision	Publication Bias	Thermal Stimulation + CT	CT Alone	Relative (95% CI)	MD or SMD (95% CI)		
Function (Overall) (Assessed with Fugl Meyer scale lower extremity; scale 0 to 34)												
2	RCT	Not serious	Not serious	Not serious	Not serious	No detected	31	32	-	MD = 6.92 points (4.36 to 9.48)	⨁⨁⨁⨁ High	CRITICAL
Motor function (Overall) (Assessed with Modified motor assessment; scale 0 to 48)												
3	RCT	Not serious	Not serious	Not serious	Not serious	Not detected	47	45	-	MD = 6.31 (5.18 to 7.44)	⨁⨁⨁⨁ High	CRITICAL
Balance (Overall) (Assessed with Berg Balance Scale; scale 0 to 56)												
3	RCT	Not serious	Very serious	Not serious	Very serious	Not detected	47	46	-	MD = 4.41 (−2.59 to 11.4)	⨁◯◯◯ Very low	CRITICAL
Walking (Overall) (Assessed with Functional Ambulation Classification; scale 0 to 5)												
3	RCT	Not serious	Very serious	Not serious	Not serious	Not detected	32	31	-	MD = 1.01 (0.33 to 1.69)	⨁⨁◯◯ Low	IMPORTANT
‡ Daily living activity (Overall) (Assessed with Barthel modified index; scale 0 to 20)												
3	RCT	Not serious	Very serious	Not serious	Not serious	Not detected	50	52	-	MD = 1.19 (−0.46 to 2.84)	⨁⨁⨁⨁ High	IMPORTANT

**CI:** confidence interval; **RCT:** randomized clinical trial; **MD:** mean difference; **Quality of evidence: High:** The research provides a very good indication of the likely effect. The probability that the effect is different is low. **Moderate:** The research provides a good indication of the likely effect. The probability that the effect is substantially different is Moderate. **Low:** The research gives some indication of the probable effect. However, the probability that the effect is substantially different is high. **Very low:** The research does not provide a reliable estimate of the probable effect. The probability that the effect is substantially different is very high. **Downgrading:** GRADE approach has four reasons for possible rate down the quality of evidence. It begins with the study designs (trials or observational studies), secondly downgrading the evidence two levels: (1) for study limitation, if the majority of studies (>50%) was rated as high risk of bias. (2) For inconsistency, if heterogeneity was greater than the accepted low level I^2^ > 40% and point estimates vary widely across studies. Confidence intervals (CIs) show minimal or no overlap; the statistical test for heterogeneity—which tests the null hypothesis that all studies in a meta-analysis have the same underlying magnitude of effect—shows a low P-value. The I^2^—which quantifies the proportion of the variation in point estimates due to among-study differences—is large). (3) For indirectness, directness was undoubled. (4) For imprecision, if the meta-analysis had a small sample size (n < 400) or very wide confidence interval. **CT**: conventional therapy; **‡**: comparison between noxious thermal stimulation + CT versus innocuous thermal stimulation + CT.

## Supplementary Materials

The following supporting information can be downloaded at: https://www.mdpi.com/article/10.3390/jcm13226937/s1, Table S1: Search strategies. Table S2: Excluded studies. Figure S1: Funnel plot for overall outcomes.
