# Supplementary material for: The Effectiveness of Thermal Stimulation Plus Conventional Therapy for Functional Recovery After Stroke: A Systematic Review and Meta-Analysis
Source: J Clin Med. 2024 Nov 18;13(22):6937. doi: 10.3390/jcm13226937 (PMC11594389; doi:10.3390/jcm13226937)
Supplement: Supplementary file 1 [file jcm-13-06937-s001.zip › jcm-3230013-supplementary.pdf]

Table S1. Search strategies

Database	Strategy	Results
MEDLINE / PubMed	#1 Search: ((((Stroke) OR (ICTUS)) OR (Post stroke)) OR (After Stroke)) OR (Hemiplegic)) OR (Hemiparetic)) OR (Cerebrovascular disorders)))<br><br>#2 Search: (((((Thermal intervention) OR (Thermal stimulation)) OR (Thermotherapy)) OR (Thermal therapy)) OR (Thermal tactile stimulation)) OR (Noxious thermal stimulation)) OR (Innocuous thermal stimulation)) OR (Thermal approach)) OR (Hyperthermia, Induced)))<br><br>#3 Search (((Randomized clinical trial) OR (Controlled clinical trial)) OR (Clinical trial)))<br><br>#4 Search (#1) AND (#2) AND (#3)	193
Scopus	TITLE-ABS-KEY ((((((((((stroke) OR (ictus)) OR (post AND stroke)) OR (after AND stroke)) OR (hemiplegic)) OR (hemiparetic)) OR (cerebrovascular AND disorders))) AND ((((randomized AND clinical AND trial) OR (controlled AND clinical AND trial)) OR (clinical AND trial)))) AND (((((((((thermal AND intervention) OR (thermal AND stimulation)) OR (thermotherapy)) OR (thermal AND therapy)) OR (thermal AND tactile AND stimulation)) OR (noxious AND thermal AND stimulation)) OR (innocuous AND thermal AND stimulation)) OR (thermal AND approach)) OR (hyperthermia, AND induced)))))	139
Web of Science	Results for ((((((((((Stroke) OR (ICTUS)) OR (Post stroke)) OR (After Stroke)) OR (Hemiplegic)) OR (Hemiparetic)) OR (Cerebrovascular disorders))) AND ((((Randomized clinical trial) OR (Controlled clinical trial)) OR (Clinical trial)))) AND ((((((((((Thermal intervention) OR (Thermal stimulation)) OR (Thermotherapy)) OR (Thermal therapy)) OR (Thermal tactile stimulation)) OR (Noxious thermal stimulation)) OR (Innocuous thermal stimulation)) OR (Thermal approach)) OR (Hyperthermia, Induced)))) (All Fields)	80
Embase	('stroke'/exp OR stroke OR 'ictus'/exp OR ictus OR 'post stroke'/exp OR 'post stroke' OR (post AND ('stroke'/exp OR stroke)) OR 'after stroke' OR (after AND ('stroke'/exp OR stroke)) OR 'hemiplegic'/exp OR hemiplegic OR hemiparetic OR 'cerebrovascular disorders'/exp OR 'cerebrovascular disorders' OR (cerebrovascular AND ('disorders'/exp OR disorders))) AND ('randomized clinical trial' OR (randomized AND ('clinical'/exp OR clinical) AND ('trial'/exp OR trial)) OR 'controlled clinical trial'/exp OR 'controlled clinical trial' OR (controlled AND ('clinical'/exp OR clinical) AND ('trial'/exp OR trial)) OR 'clinical trial'/exp OR 'clinical trial' OR (('clinical'/exp OR clinical) AND ('trial'/exp OR trial))) AND ('thermal intervention' OR (thermal AND ('intervention'/exp OR intervention)) OR 'thermal stimulation'/exp OR 'thermal stimulation' OR (thermal AND ('stimulation'/exp OR stimulation)) OR 'thermotherapy'/exp OR thermotherapy OR 'thermal therapy'/exp OR 'thermal therapy' OR (thermal AND ('therapy'/exp OR therapy)) OR 'thermal tactile stimulation' OR (thermal AND tactile AND ('stimulation'/exp OR stimulation)) OR 'noxious thermal stimulation' OR (noxious AND thermal AND ('stimulation'/exp OR stimulation)) OR 'innocuous thermal stimulation' OR (innocuous AND thermal AND ('stimulation'/exp OR stimulation)) OR 'thermal approach' OR (thermal AND approach) OR 'hyperthermia, induced'/exp OR 'hyperthermia, induced' OR (('hyperthermia,'/exp OR hyperthermia,) AND induced))	344
CINAHL complete	((((((((((Stroke) OR (ICTUS)) OR (Post stroke)) OR (After Stroke)) OR (Hemiplegic)) OR (Hemiparetic)) OR (Cerebrovascular disorders))) AND ((((Randomized clinical trial) OR (Controlled clinical trial)) OR (Clinical trial)))) AND ((((((((((Thermal intervention) OR (Thermal stimulation)) OR (Thermotherapy)) OR (Thermal therapy)) OR (Thermal tactile stimulation))	7

	OR (Noxious thermal stimulation)) OR (Innocuous thermal stimulation)) OR (Thermal approach)) OR (Hyperthermia, Induced))))	
SPORTDiscus	((((((((((Stroke) OR (ICTUS)) OR (Post stroke)) OR (After Stroke)) OR (Hemiplegic)) OR (Hemiparetic)) OR (Cerebrovascular disorders))) AND ((((Randomized clinical trial) OR (Controlled clinical trial)) OR (Clinical trial)))) AND ((((((((((Thermal intervention) OR (Thermal stimulation)) OR (Thermotherapy)) OR (Thermal therapy)) OR (Thermal tactile stimulation)) OR (Noxious thermal stimulation)) OR (Innocuous thermal stimulation)) OR (Thermal approach)) OR (Hyperthermia, Induced))))	3
Epistemonikos	((((((((((Stroke) OR (ICTUS)) OR (Post stroke)) OR (After Stroke)) OR (Hemiplegic)) OR (Hemiparetic)) OR (Cerebrovascular disorders))) AND ((((Randomized clinical trial) OR (Controlled clinical trial)) OR (Clinical trial)))) AND ((((((((((Thermal intervention) OR (Thermal stimulation)) OR (Thermotherapy)) OR (Thermal therapy)) OR (Thermal tactile stimulation)) OR (Noxious thermal stimulation)) OR (Innocuous thermal stimulation)) OR (Thermal approach)) OR (Hyperthermia, Induced))))	14
LILACS	((((((((((Stroke) OR (ICTUS)) OR (Post stroke)) OR (After Stroke)) OR (Hemiplegic)) OR (Hemiparetic)) OR (Cerebrovascular disorders))) AND ((((Randomized clinical trial) OR (Controlled clinical trial)) OR (Clinical trial)))) AND ((((((((((Thermal intervention) OR (Thermal stimulation)) OR (Thermotherapy)) OR (Thermal therapy)) OR (Thermal tactile stimulation)) OR (Noxious thermal stimulation)) OR (Innocuous thermal stimulation)) OR (Thermal approach)) OR (Hyperthermia, Induced))))	3
PEDro	Abstract & Title: thermal stimulation<br>Therapy: Electrotherapies, heat, cold<br>Subdiscipline: Neurology<br>Method: Clinical trial<br>Title Only: STROKE<br>Match all search terms (AND)	10
**Total**		**793**

31 August 2024

Table S2. Excluded studies

N°	Reference	Reason
1	Abbas M, Ansari AN, Nayab M, Afzal U, Abalkhai AA.Efficacy of Hot and Dry Bath (Hammam-i-Har-Yabis) in Activities of Daily Living for Management of Post Stroke Hemiplegia: An Exploratory Clinical TrialJ Clin of Diagn Res.2022; 16(3):KC05-KC08. https://www.doi.org/10.7860/JCDR/2022/53459/16121	No randomized clinical trial
2	Matsumoto S, Shimodozono M, Etoh S, Noma T, Uema T, Ikeda K, Miyara K, Tanaka N, Kawahira K. Anti-spastic effects of footbaths in post-stroke patients: a proof-of-principle study. Complement Ther Med. 2014 Dec;22(6):1001-9. doi: 10.1016/j.ctim.2014.09.006. Epub 2014 Sep 30. PMID: 25453520.	No noxious thermal stimulation
3	Moraes, Jéssica Camila de et al. O aquecimento e o resfriamento terapêutico melhoram a amplitude de movimento imediatamente após a aplicação dos recursos na condição de espasticidade após acidente vascular encefálico. SALUSVITA, Bauru, v. 36, n. 2, p. 463-74, 2017	No noxious thermal stimulation
4	Chen SC, Hsu MJ, Kuo YT, Lin RT, Lo SK, Lin JH. Immediate effects of noxious and innocuous thermal stimulation on brain activation in patients with stroke. Medicine (Baltimore). 2020 Feb;99(9):e19386. doi: 10.1097/MD.0000000000019386. PMID: 32118788; PMCID: PMC7478460.	No functional outcomes
5	Tai I, Lai CL, Hsu MJ, Lin RT, Huang MH, Lin CL, Hsieh CL, Lin JH. Effect of thermal stimulation on corticomotor excitability in patients with stroke. Am J Phys Med Rehabil. 2014 Sep;93(9):801-8. doi: 10.1097/PHM.0000000000000105. PMID: 24800718.	No functional outcomes

Figure S1. Funnel plot for overall outcomes

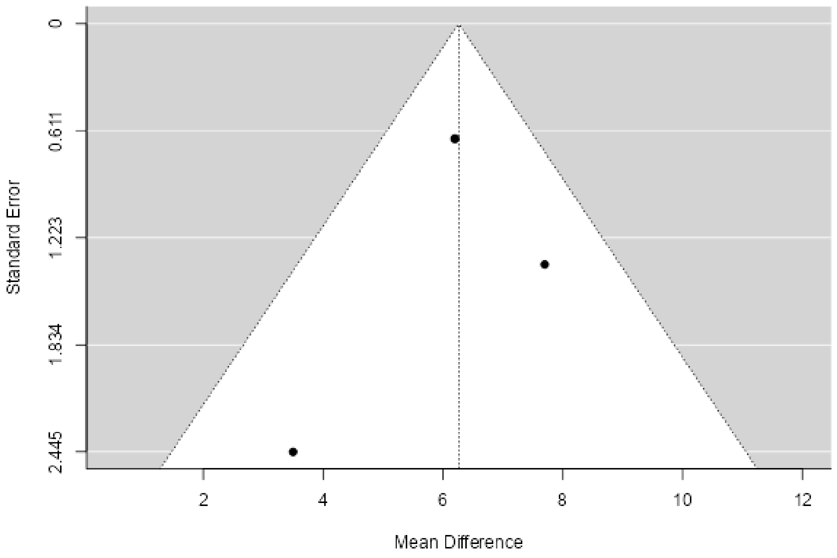

*** Egger test: *p=0.532***
